# Changes in Structural and Rheological Properties of Guar Gum Particles in Fluidized-Bed Agglomeration: Effect of Sucrose Binder Concentration

**DOI:** 10.3390/foods11010073

**Published:** 2021-12-29

**Authors:** Donghyeon Lee, Byoungseung Yoo

**Affiliations:** Department of Food Science and Biotechnology, Dongguk University-Seoul, Goyang 410-820, Korea; ldhdongdong@gmail.com

**Keywords:** fluidized-bed agglomeration, FTIR spectrophotometry, crystallinity determination, dispersion behavior, particle size

## Abstract

Fluidized-bed agglomeration (FBA) is known to modify the structure and rheology of food powders. In this study, guar gum (GG) powders with various concentrations of sucrose binder (0%, 10%, 20%, or 30%) were subjected to fluidized-bed agglomeration. Subsequently, changes in the characteristics of the GG powders were evaluated by using scanning electron microscopy (SEM), Fourier-transform infrared spectroscopy (FTIR), X-ray diffraction (XRD), particle size distribution (PSD) analysis, and rheological and dispersibility measurements. SEM images and FTIR spectra revealed surface morphology changes and structural modification, respectively, in the original GG powder after FBA, although the changes observed in FTIR spectra were only slightly dependent on sucrose concentration at low concentrations (0–20%). XRD patterns confirmed that the crystallinity of the GG powder was affected by FBA, but not greatly so by binder concentration. The PSD results showed that the GG particle size was increased by FBA and there was a clear relationship between sucrose concentration (10–30%) and mean particle size. The rheological behavior and dispersibility of GG (properties that are known to be affected by the structure of a powder) were also influenced by sucrose concentration. To sum up, FBA and the concentration of sucrose binder used can serve as factors for modifying GG powder.

## 1. Introduction

The fluidized-bed agglomeration (FBA) process is started by placing the primary powder in the container of the agglomeration chamber and fluidizing it by using an upward hot air stream. A water-based low-viscosity liquid, called a binder, is then sprayed on the powder flowing through the agglomeration chamber. Powder particles coalesce during the spraying period (agglomeration) but also disintegrate due to collisions with other powder particles or the chamber wall (attrition). The FBA process modifies various characteristics of food powders, including the outer structure, size distribution, flowability, density, wettability, and dissolution behavior [1,2,3]. The FBA process is also known to modify the rheological properties of the original material [4].

The FBA process is widely used in the food industry to modify food powders. Dairy-based powders (e.g., skim and whole milk powders, milk/whey protein isolate powder, dairy-based multi-component powders, etc.), fruit and vegetable powders (e.g., soymilk powder, cocoa powder, bayberry powders, spinach powder, carrot concentrate powder, etc.), and cereal powders (e.g., durum wheat semolina and corn, manioc, amaranth, buckwheat, and quinoa flour, etc.) are primarily used as materials in the food industry [5]. Recently, the FBA process has been conducted not only on food powders derived from commercial foodstuffs, but also on gum powders mainly used as thickeners, gelling agents, emulsifiers, and stabilizers.

Among the food gums, a galactomannan-type hydrocolloid consisting of a polymannose backbone and attached galactose branches is of great interest. Specifically, β-d-mannopyranosyl units are linked to each other with β-(1→4) linkages while α-d-galactopyranosyl units are attached to the main chain. These structures contain numerous exposed hydroxyl groups in the galactomannan molecule, which enables them to easily attach to other gums or water molecules via hydrogen bonds [6]. Especially, galactomannans attach in solution via hydrogen bonds between the hydroxyl groups of each molecule, and this molecular interaction increases the viscosity of the solution [7]. The differences in the properties (e.g., solubility, viscosity, and gel-forming ability) of galactomannans compared to other polysaccharides are determined by differences in their side-chain distributions [8,9]. Among the various galactomannans, guar gum (GG) is regarded as an interesting material by the food industry. In GG, mannose and galactose are present at a ratio of 1.8:1 (comparably smaller than other galactomannans such as locust bean gum (LBG) and tara gum (TG)) with galactose side units evenly distributed on the polymannose main chain, which is rarely seen in other galactomannans [10]. This structural difference changes the physical properties of GG compared to other galactomannans that have unevenly distributed galactose units. Besides its physical properties, GG also has rheological advantages over other galactomannans [11] as even a small amount of GG can effectively change the viscosity of a product. Therefore, GG is favored as a thickening agent and, in particular, it is used as the main gum component of commercial instant food thickeners for people with dysphagia [12]. However, since GG is commercially distributed and used in the form of a powder, it is difficult to handle due to problems such as poor dispersibility and dust emission [13]. Moreover, the excessively high viscosities obtained when using GG limit its use, and so proper modification of GG is required [14]. These limitations can be overcome by applying the FBA process to GG.

According to several researchers, the three stages (coating, agglomeration, and attrition) of the fluidized-bed process are determined by numerous factors. These include the original shape of the powder, the type and concentration of the binder solution, the fluidizing air velocity inside the agglomeration chamber, the nozzle air spraying pressure, and the binder flow rate [4,15,16,17,18,19]. In food systems, a water-based solution (sugars, food gums, and starches) is commonly used as a binder. It is also known that the agglomeration of particles greatly depended on the properties of binder solution, particularly the type of binder and the concentration of binder solution [20]. In particular, Lee and Yoo [16] and Lee et al. [21] applied various sugar binder solutions at the same concentration to food gums and concluded that the addition of sugar binder in the agglomeration process was beneficial and improved the physical properties of the fine gum powders by greatly influencing the structure of the primary particles. They also found that the physical properties of agglomerated gums were greatly influenced by the type of sugar binder. However, understanding the relationship between the sugar binder concentration and the structural and rheological properties of agglomerated gum powder is crucial for applying the process to yield the required and desirable product. Especially, knowledge about the structure modification of agglomerated gum powder prepared with different sugar concentrations is very important since the internal and external structures greatly affect the properties of the powder, such as its flowability, dispersibility, and rehydratability.

Researchers currently apply several methods, including scanning electron microscopy (SEM), Fourier-transform infrared (FTIR) spectroscopy, X-ray diffraction (XRD), and dynamic rheometry, to investigate the structure of small particles. Native gum powders, such as GG and xanthan gum (XG), have been investigated via these methods [22,23]. However, no attempt has been made to study the structural and rheological properties of agglomerated GG powders prepared with different sucrose binder concentrations. Therefore, in this study, GG was agglomerated with a sucrose binder at various concentrations (0%, 10%, 20%, and 30%), and the main objective of this research was to investigate the effect of sucrose binder with different concentrations on the structural (SEM, FTIR, and XRD) and rheological (steady shear and dynamic shear) properties of the agglomerated GG powders. Besides, their dispersion behavior and dispersion stability, which are possibly related to the structural properties of the GG agglomerates, were also examined using a turbidimeter.

## 2. Materials and Methods

### 2.1. Materials

GG (Habgen Guargums Ltd., Karachi, Pakistan) in powder form was used as the raw material. Binder solutions were prepared by dissolving sucrose (Samyang Co., Ltd., Seongnam, Korea) in distilled water at concentrations ranging from 0% to 30% (*w*/*w*). Each solution was stirred for 20 min and then left overnight to complete hydration. In the agglomeration process, 1500 g of GG was fluidized by using an inlet hot air stream at 75 ± 1.0 °C. Next, the binder solution was fed via a peristaltic pump and sprayed through a two-fluid nozzle into the granulation chamber (volume: 10 L) (Fluid Bed Lab System, Dae Ho Technology Co., Ltd., Hwaseong, Korea) at a speed of 20 mL/min for 50 min according to the procedure of Lee et al. [21]. During spraying, the temperature of the powder was kept at 53 ± 1.0 °C. The blower and damper were adjusted to 70% and 30%, respectively. After spraying, the product was dried and cooled in the same chamber by fluidizing the air stream at room temperature for 10 min. The moisture contents of all agglomerated samples were in the range of 4.00–4.28%.

### 2.2. Particle Morphology

SEM (Hitachi S-3000 N SEM, Hitachi Ltd., Tokyo, Japan) was used to visualize the surface morphology of the powders. Powders were spread on a disk-shaped aluminum stub with a diameter of 4 cm, then coated with platinum–palladium under vacuum conditions [12]. Subsequently, SEM was conducted at 20 kV to acquire images at 70× and 100× magnification.

### 2.3. Chemical Structural Analysis

Fourier-transform infrared spectroscopy (FTIR) was used to characterize the chemical structure of the powders. 1 mg of sample and 100 mg of potassium bromide were combined and ground by using a pestle and mortar. Next, the mixture was compressed to form a pellet, which was subsequently loaded into an FTIR spectrophotometer (Cary 610/660, Agilent Technologies Inc., Santa Clara, CA, USA) at room temperature. FTIR spectra of the powders were obtained in the wavelength range of 4000–400 cm^−1^ with a resolution of 4 cm^−1^ and then analyzed to investigate the chemical structures of the agglomerates [22].

### 2.4. Crystallinity Determination

To determine any differences in crystallinity between the powder samples, they were subjected to XRD (Ultima IV, Rigaku Corp, Tokyo, Japan) with angular intervals ranging from 10–70° (2θ range), a scanning speed of 3°/min, steps of 0.02° and 2 s per step at room temperature [24].

### 2.5. PSD

Particle size (D_10_, D_50_, and D_90_) and uniformity (span) of the powders were determined through laser diffraction methods using a Malvern Mastersizer 3000E (Malvern Instruments Ltd, Worcestershire, UK) [21]. From the measurements, D_10_, D_50_, and D_90_ are computed to investigate differences in the average particle sizes of the powders depending on the binder concentration. The span index was calculated by using the following equation:

Span = (D_90_ − D_10_)/D_50_ (1)

where D_10_, D_50_, and D_90_ are the particle diameters at 10%, 50%, and 90% in the cumulative size distribution, respectively.

### 2.6. Rheological Properties

Before rheological measurements, solutions were prepared by dissolving 1.0 g of agglomerated GG in 100 mL of distilled water. Each solution was stirred for 4 h and left overnight to allow the powder to fully hydrate. After preparation of the solutions, their flow and dynamic rheological properties were measured by using a Haake RheoStress 1 rheometer (Haake GmbH, Karlsruhe, Germany) according to the procedure of Lee and Yoo [12]. In both measurements, plate–plate geometry with a 35 mm diameter was used, and the gap between the plates was set as 500 μm. To investigate the flow rheological properties of the agglomerates, the shear stress of the solution was measured for a shear rate range of 0.1–100 s^−1^. The experimental results were applied to the power-law equation as follows:

(2) $$\sigma =K\times {\dot{\gamma }}^{n}$$

where *σ* is the shear stress (Pa), $\dot{\gamma }$ is the shear rate (s^−1^), and *n* is the flow behavior index, after which consistency index (K) and the apparent viscosity at 50 s^−1^ (η_a,50_) were calculated. Dynamic rheological behaviors of the agglomerates were investigated by frequency sweep test in the angular frequency range of 0.63–62.8 rad·s^−1^ with 2% strain, which is in linear viscoelasticity limit of the sample. From the measurement, storage (or elastic) modulus (G′), loss (or viscous) modulus (G″), and loss tangent (tan δ) of agglomerate at 6.28 rad·s^−1^ were calculated by Haake Rheowin software (ver. 4.50.0003) and used to investigate viscoelasticity difference between the agglomerates.

### 2.7. Dispersion Behavior

A turbidimeter (AQ4500, Thermo Fisher Scientific Inc., Waltham, MA, USA) was used to evaluate the dispersion behavior of the agglomerated GG powder (the dispersion behavior of the non-agglomerated GG powder could not be measured because of lump formation) [12]. First, 0.3 g of powder was dispersed in 100 mL of water at room temperature by stirring at 350 rpm. Subsequently, the turbidity of the dispersion was measured after stirring for 10, 20, 30, 60, 90, 150, and 180 s.

### 2.8. Statistical Analysis

All experiments were measured 3 times and each experimental data item is reported as the mean and standard deviation. Duncan’s multiple range test based on analysis of variance (ANOVA) was used to reveal statistically significant differences among the mean values (*p* < 0.05).

## 3. Results and Discussion

### 3.1. Particle Morphology

**Figure 1:**
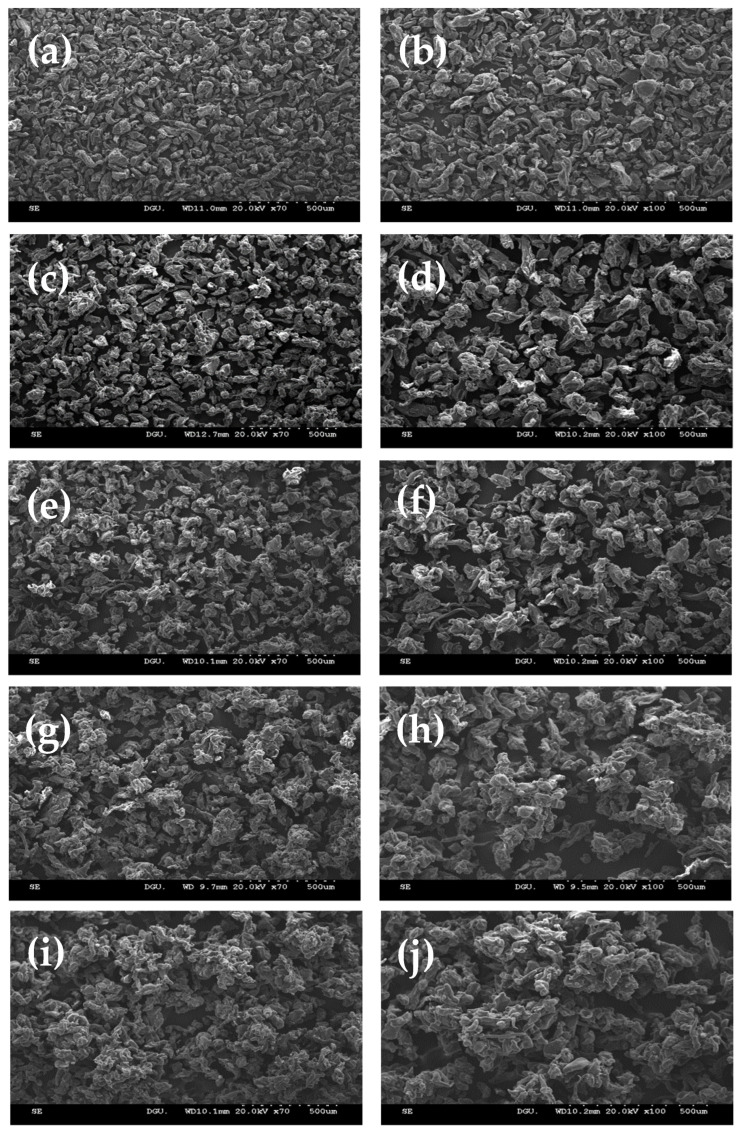
Respective low-magnification (70×) and high-magnification (100×) SEM micrographs of (**a**,**b**) non-agglomerated and agglomerated guar gum (GG) powders with various concentrations of sucrose binder: (**c**,**d**) GG—0%, (**e**,**f**) GG—10%, (**g**,**h**) GG—20%, (**i**,**j**) GG—30%.

The morphologies of both the non-agglomerated and agglomerated GG powders were visualized by using SEM, as illustrated in Figure 1. The particle size was larger after agglomeration; thus, the non-agglomerated GG comprised small and compact particles with a smooth surface, whereas the agglomerated GG with sucrose binder comprised large and irregularly shaped particles with wrinkled and porous surfaces. It was also confirmed that the size of the particles was affected by the concentration of the sucrose binder, showing that the highest concentration of the binder yielded the largest particles. This indicates that the addition of sucrose binder solution with high concentrations appears to form large particles with dense and porous structures due to the strong interactions between GG particles. In addition, the irregularly shaped particles in agglomerated GG experience friction between them, which affects the movement of the particles and their flowability [25]. Szulc and Lenart [1] and Jinapong et al. [26] reported that the binder concentration used during the agglomeration process is a possible factor for determining their surface morphology.

Besides particle growth and surface roughness, the empty portion of the powder can also be partially seen in the SEM images. Agglomerated GG, especially with a higher concentration of binder, contained more porous spaces in the particles through which the penetration of solvents or dispersing medium can occur when the powders are dispersed in liquids. This causes particles with high porosity to wet more quickly. From these results, it can be concluded that applying a binder at a high concentration is suitable for manufacturing agglomerated GG powder from the original material via an FBA process because the fast dissolution or dispersion of food powders is required by the food industry.

### 3.2. Chemical Structural Analysis

**Figure 2:**
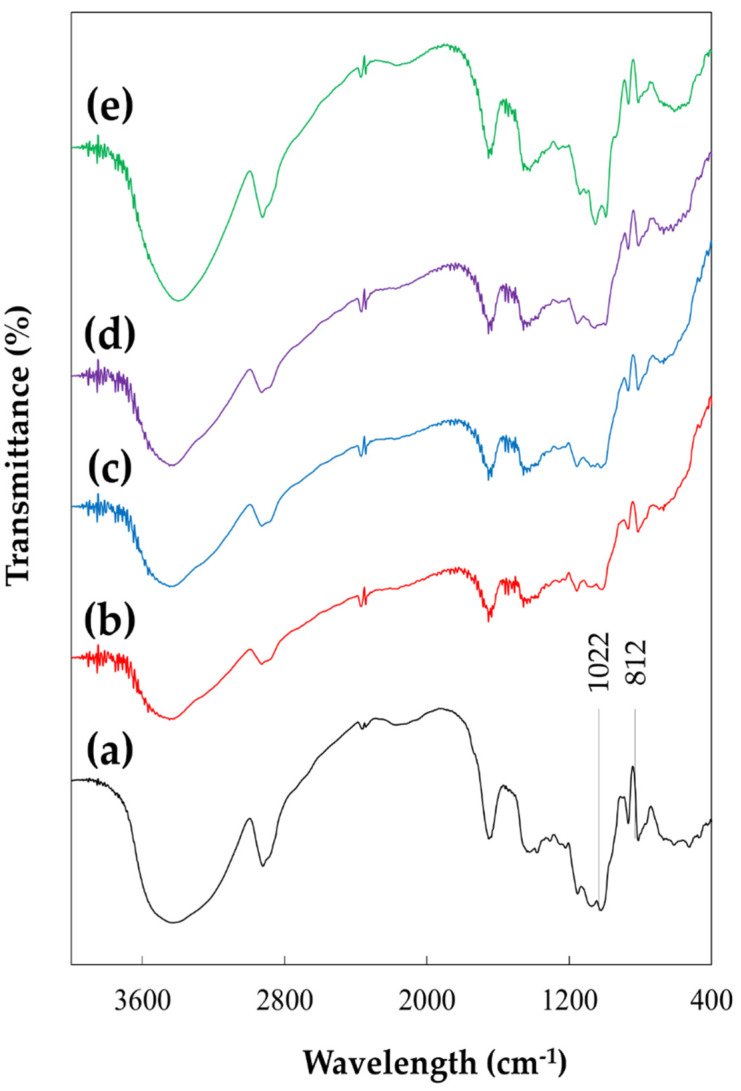
FTIR spectra of (**a**) non-agglomerated and agglomerated guar gum (GG) with various concentrations of sucrose binder: (**b**) GG—0%, (**c**) GG—10%, (**d**) GG—20%, and (**e**) GG—30%.

Figure 2 shows the FTIR spectra of non-agglomerated and agglomerated GG. Differences in peak distribution occurred at 3600–2800 cm^−1^, 1022 cm^−1^, and 812 cm^−1^. According to Cerqueira et al. [23] and Mudgil et al. [24], peaks at 3600–2800 cm^−1^ indicate the presence of hydroxyl groups and methyl groups, and the peak at 1022 cm^−1^ represents a bond between C-O and C-O-H, which can be commonly seen in the structure of carbohydrates.

When compared to agglomerated GG, the intensity of peaks at those wavelengths was stronger in non-agglomerated GG, meaning that there is the possibility of a breakdown of the primary structure of GG during the FBA process. There were no clear differences between the peak intensities for agglomerates with a sucrose concentration of 0–20%, but increased peak intensities were observed in the agglomerate with 30% sucrose due to the excessive amount of sucrose molecules added to GG during the FBA process.

According to Duarte et al. [27], a high concentration of sucrose resulted in the appearance of peaks in the wavelength range of 1150–950 cm^−1^. Meanwhile, the peak at 812 cm^−1^ (indicating the presence of β-D-mannopyranose units was weaker for agglomerated GG than non-agglomerated GG, meaning that the main polymannose chain had been modified [22]. Moreover, the decreased intensity could be due to the loss of ring structure resulting from reactions that caused the ring-opening of the monosaccharides that comprise GG. From these observations, it can be concluded that the primary structure of GG was clearly modified during the FBA process, especially the polymannose backbone structure. Hence, low sucrose concentrations (0–20%) could have been consumed to modify the structure of the GG backbone whereas a higher concentration of sucrose could have further affected the structure by attaching to the GG backbone.

### 3.3. Crystallinity

**Figure 3:**
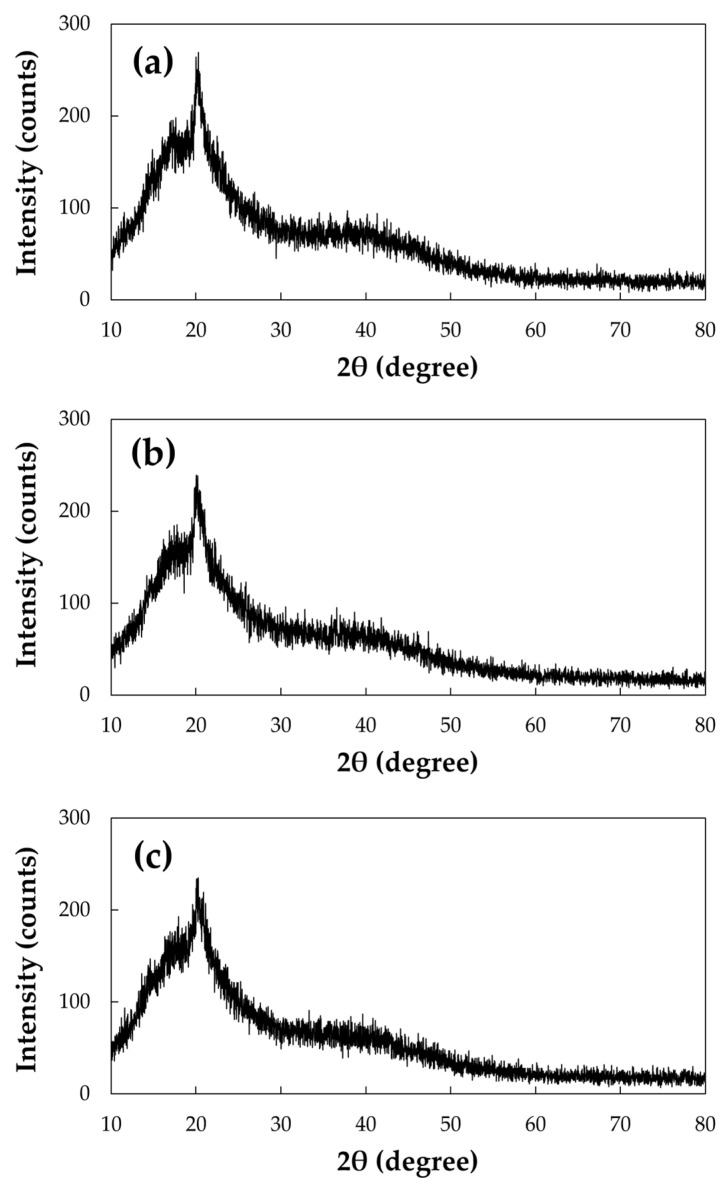
XRD patterns of (**a**) non-agglomerated and agglomerated guar gum (GG) with water (GG—0%) (**b**) and sucrose binder (GG—30%) (**c**).

Figure 3 illustrates XRD patterns of the non-agglomerated and agglomerated GG powders prepared with water and sucrose (30%). All of the patterns display irregular peaks in all areas (especially the wide peak at 2θ of 20°), which indicates the amorphous nature of the powders. In particular, there were distinct differences between the non-agglomerated and agglomerated GG powders. The intensity of patterns in the wide peak was higher for the non-agglomerated GG, which means that it had relatively higher crystallinity than the agglomerated GG. From the FTIR results, the mannopyranose structure was shown to be broken by the FBA process, which is a possible factor for the decrease in crystallinity in the agglomerated GG powder. This could have resulted in better penetration by water, which would explain the faster dissolution of agglomerated GG than non-agglomerated GG [28]. However, there were no clear differences between the GG agglomerates regardless of sucrose concentrations, which means that although the crystallinity of the native GG powder was greatly influenced by the FBA process, the sucrose concentration had no effect.

### 3.4. PSD Analysis

**Figure 4:**
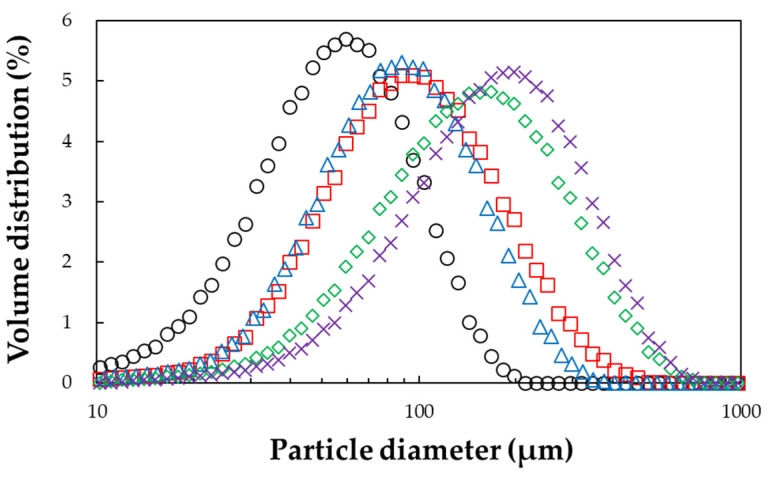
PSD of non-agglomerated and agglomerated guar gum (GG) with various concentrations of sucrose binder: (_◯_) non-agglomerated GG, (□) GG—0%, (△) GG—10%, (◇) GG—20%, and (×) GG—30%.

The particle diameters (D_10_, D_50_, and D_90_) and uniformity (span) of the GG powders are reported in Table 1 and Figure 4. It was found that the particle size increased with an increase in sucrose concentration, except for the GG agglomerate with 10% sucrose, which was actually smaller than the agglomerate with 0% sucrose. In comparison with non-agglomerated GG powder, the particle size of the agglomerated powder increased (as confirmed via the SEM images), which has previously been observed when native gum powders are subjected to FBA [21]. The results of former studies suggest that the particle size of gum powders grows proportionally with the concentration or viscosity of the binder solution used in the agglomeration process [12,16]. In general, coating and agglomerating powders with binder solutions at various concentrations may require different times to complete the process, resulting in differences between products [15]. However, we found no consistent relationship between span value and sucrose concentration. From these observations, it can be clarified that the sucrose binder concentration has a distinct relationship with particle size. Therefore, the careful selection of binder concentration is required for developing model powders.

### 3.5. Rheological Properties

In combination with FTIR, investigating the rheological properties of a polysaccharide dispersion is an effective tool for estimating the plausible existence of or changes in molecular interactions between polysaccharides [29]. In this study, the rheological properties of non-agglomerated and agglomerated GG were investigated from two perspectives:steady shear and dynamic shear rheological properties. The shear stress (*σ*) versus shear rate ($\dot{\gamma }$) data were well fitted with the power-law model with a high coefficient of determination (R^2^ = 0.98–0.99). From the results of the steady shear test, non-agglomerated GG powder showed relatively higher viscosity values (η_a,50_ and K) (compared to GG agglomerated with higher concentrations (20% and 30%) of sucrose binder (Table 2). The η_a,50_ and K values also decreased with an increase in sucrose concentration from 10% to 30%. This indicates that sucrose molecules introduced into the agglomeration chamber as a binder covered the non-substituted area in the GG molecules, which could have interfered with interactions between the GG molecules and thereby decreased the viscosity. Moreover, the relationship between the pseudoplastic behavior of agglomerated GG and binder concentration was also observed based on the difference in *n* values. Agglomerated GG without sucrose binder had the lowest *n*, even lower than that of non-agglomerated GG, indicating that it was less sticky than native GG. It is well known that a high *n* value results in a sticky mouthfeel, whereas a low *n* value results in a smooth mouthfeel [30]. However, *n* values of agglomerated GG increased with an increase in sucrose, with similar values for sucrose concentrations of 10–20% and the highest value at 30% concentration. This means that FBA modifies the pseudoplastic behavior of GG, depending on the concentration of sucrose binder in the range of 0–30%. Generally, the degree of pseudoplasticity of gum materials can be determined from their conformation and molecular weight, showing the possibility of structural change in GG. The increased *n* values also mean that the addition of sucrose binder can confer a sticky mouthfeel to the agglomerated GG powder.

**Figure 5:**
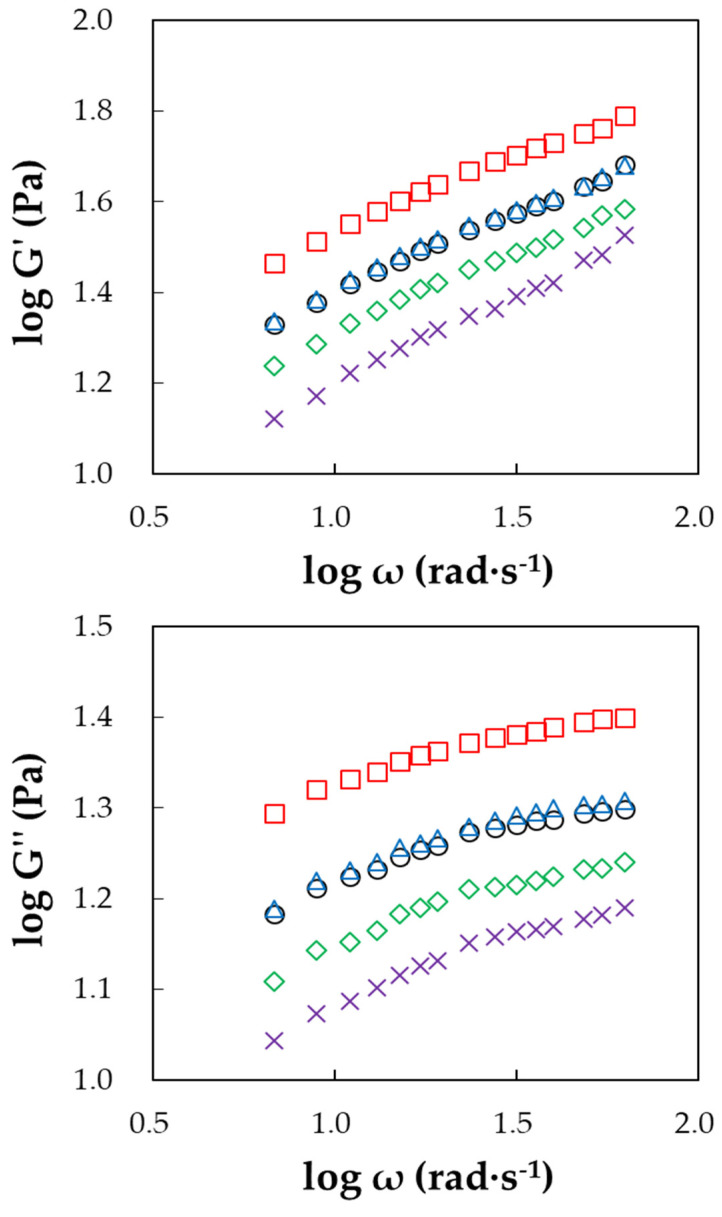
Plots of log G’ and log G’’ versus log ω for non-agglomerated and agglomerated guar gum (GG) with various concentrations of sucrose binder: (○) non-agglomerated GG, (□) GG—0%, (△) GG—10%, (◇) GG—20%, and (×) GG—30%.

The results of the dynamic shear test confirm that the viscoelastic properties of GG were also affected by the FBA process and variation in the sucrose concentration. Both G’ and G” values decreased with the increase in binder concentration from 0 to 30 % (Table 2 and Figure 5); such great changes in the dynamic moduli values could have resulted from difficulties with chain organization recovery. This also suggests differences in molecular interaction behavior inside the prepared GG dispersions that could have originated from structural modification [31]. In all cases, G’ was larger than G”, which means that both non-agglomerated and agglomerated GG displayedelastic behavior rather than viscous behavior. Moreover, the gradual increase in tan δ values with increasing sucrose concentration implied that the samples became more viscous. These results indicate that the addition of sucrose decreased the solid character of agglomerated GG powder and effectively contributed to the viscous properties. From these observations, the steady and dynamic rheological properties of non-agglomerated and agglomerated GG were found to have been greatly affected by both the agglomeration process and sucrose binder concentration.

### 3.6. Dispersion Behavior

**Figure 6:**
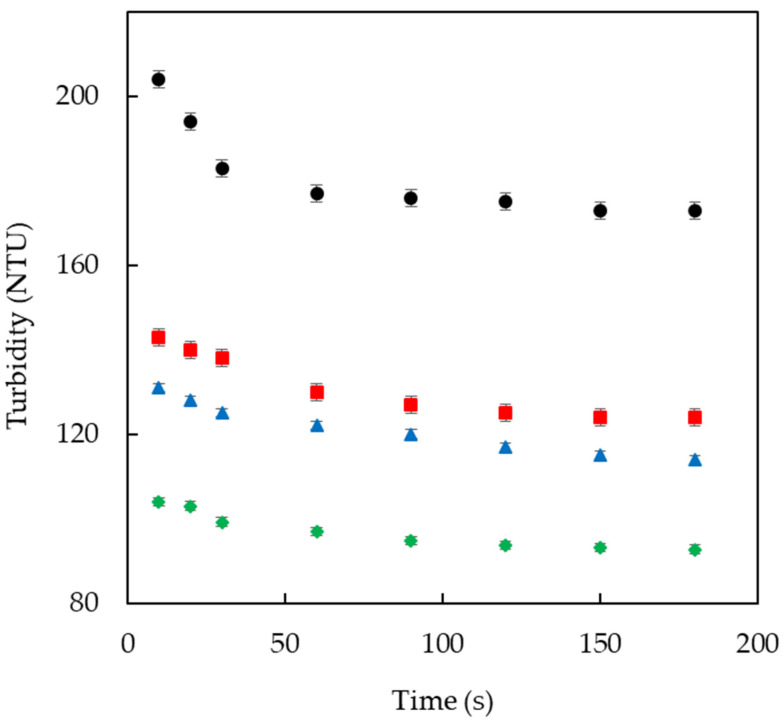
Plots of turbidity versus dissolution time of agglomerated guar gum (GG) with various concentrations of sucrose binder: (●) GG—0%, (_■_) GG—10%, (▲) GG—20%, and (◆) GG—30%.

The dispersion behavior of agglomerated GG was evaluated by analyzing changes in turbidity, as shown in Figure 6. In general, gum powders first swell and then become successively smaller as they disperse [32]. However, in the current study, there was no increase in dispersion turbidity, which means that the volume increase of the GG powder in water was almost instantaneous. Thus, the FBA process was effective at shortening the wetting period of the GG powder. This is related to the results of Su et al. [28] who stated that shortened swelling time has a connection with the intermolecular interaction and structural property of gum powder. Structural differences induced by agglomerating the GG powder may have changed interactions between the molecules, thereby reducing the time required for the powder to swell and dissolve in water.

It can be seen that difference in turbidity value between the dispersion stirred for 10 s and 180 s was inversely proportional to the concentration of sucrose binder. This is related to the stability of the dispersion, which is determined by the structural properties of the powder. In dispersed powder solutions, the stability is affected by structural properties, such as surface morphology [33]. Besides stability, the sucrose concentration can influence the dissolution rate of GG agglomerates since the hydrophilic nature of sucrose leads to hydrophilic solid bridges, easing water penetration and hastening powder dispersion, as reported by Ji et al. [34].

## 4. Conclusions

The structural and rheological properties of agglomerated GG powder prepared with sucrose as a binder solution in the FBA process were studied via diverse methods. These properties were compared between agglomerated GG powders at different sucrose binder concentrations. The surface morphology, FTIR spectra, XRD pattern, dispersibility, and PSD of GG powders were greatly influenced by FBA. In particular, the agglomerates fabricated with a higher concentration of sucrose binder had rougher surfaces and higher porosity. The higher sucrose binder concentration also influenced the dissolution rate of GG agglomerates due to the hydrophilic nature of sucrose, which leads to hydrophilic solid bridges, easing water penetration and accelerating powder dispersion. From a rheological perspective, a clear difference in both steady and dynamic shear properties between non-agglomerated and agglomerated GG was observed, and the dependence of the rheological properties on binder concentration was also confirmed by the greater structural changes in agglomerated GG powders prepared with higher binder concentrations. From these observations, it was evident that the molecular interactions of GG in water were affected by FBA and increasing sucrose binder concentration. These results demonstrate that the structural and rheological properties of agglomerated GG powders prepared with a sucrose binder solution are greatly influenced by the binder concentration. In addition, these results suggested that the careful selection of sucrose binder concentration is required for developing the agglomerated gum powders because the binder concentration could lead to large changes in the structural and rheological characteristics of gum powder. Knowledge of the specific structural and rheological properties of GG agglomerated with different sucrose binder concentrations may be useful for the manufacturing of agglomerated gum powders as a thickening agent in food systems. Further studies on various types of sugar-based binder solutions with different concentrations are needed to expand the results of this study.

## Figures and Tables

**Table 1:** PSD of non-agglomerated and agglomerated guar gum (GG) powders with various concentrations of sucrose binder.

Sample	Sucrose Concentration (%)	D_10_ (μm)	D_50_ (μm)	D_90_ (μm)	Span (-)
Non-agglomerated GG		25.0 ± 0.0 ^e^	56.7 ± 0.1 ^e^	109.4 ± 0.5 ^e^	1.48 ± 0.01 ^e^
Agglomerated GG	0	43.5 ± 0.1 ^c^	97.6 ± 0.2 ^c^	212.3 ± 0.7 ^c^	1.73 ± 0.01 ^b^
	10	41.6 ± 0.1 ^d^	89.5 ± 0.4 ^d^	179.2 ± 1.1 ^d^	1.54 ± 0.00 ^d^
	20	61.7 ± 0.1 ^b^	152.2 ± 0.6 ^b^	331.4 ± 0.1 ^b^	1.77 ± 0.01 ^a^
	30	74.3 ± 0.2 ^a^	180.3 ± 0.5 ^a^	368.1 ± 1.5 ^a^	1.63 ± 0.01 ^c^

Each value is the mean of three measurements ± SD. The mean values with different lowercase letters (a–e) within each column are significantly different (*p* < 0.05).

**Table 2:** The magnitude of power-law model parameters (η_a,50_, K, and *n*) and dynamic rheological parameters (G’, G”, and tan δ) of non-agglomerated and agglomerated guar gum (GG) with various concentrations of sucrose binder.

Sample	Sucrose Concentration (%)	η_a,50_ (Pa·s)	K (Pa·s^n^)	*n* (-)	G’ (Pa)	G” (Pa)	tan δ (-)
Non-agglomerated GG		1.00 ± 0.01 ^c^	13.31 ± 0.15 ^b^	0.34 ± 0.00 ^c^	21.20 ± 0.01 ^c^	15.73 ± 0.06 ^c^	0.74 ± 0.00 ^c^
Agglomerated GG	0	1.18 ± 0.02 ^a^	17.20 ± 0.15 ^a^	0.31 ± 0.01 ^d^	29.10 ± 0.21 ^a^	20.17 ± 0.07 ^a^	0.69 ± 0.01 ^d^
	10	1.07 ± 0.03 ^b^	13.54 ± 0.10 ^b^	0.36 ± 0.01 ^b^	21.74 ± 0.09 ^b^	15.97 ± 0.02 ^b^	0.73 ± 0.01 ^c^
	20	0.82 ± 0.01 ^d^	10.24 ± 0.08 ^c^	0.36 ± 0.01 ^b^	17.33 ± 0.13 ^d^	13.10 ± 0.14 ^d^	0.76 ± 0.01 ^b^
	30	0.66 ± 0.01 ^e^	7.09 ± 0.04 ^d^	0.39 ± 0.00 ^a^	13.14 ± 0.02 ^e^	11.34 ± 0.11 ^e^	0.86 ± 0.01 ^a^

Each value is the mean of three measurements ± SD. The mean values with different lowercase letters (a–e) within each column are significantly different (*p* < 0.05).

## Data Availability

All the results showed in the manuscript could be requested to the corresponding author who would provide them.
